# Quantitative Control of Transposable Elements: From Genome Plasticity to Immune Regulatory Circuits

**DOI:** 10.1002/cbf.70252

**Published:** 2026-07-01

**Authors:** Irving Jesús Reyes‐Barragán

**Affiliations:** ^1^ Instituto Politécnico Nacional Escuela Superior de Medicina (ESM‐IPN) Ciudad de México México; ^2^ Instituto Nacional de Medicina Genómica (INMEGEN) Ciudad de México México

## Abstract

Transposable elements (TEs) constitute nearly half of the human genome and are increasingly recognized as context‐dependent regulators of genome function rather than passive repetitive DNA. This Review synthesizes classical and recent evidence on TE biology, including TE classification, mechanisms of mobilization, host restriction pathways, insertional mutagenesis, and contributions to gene regulation. We emphasize that TE activity operates across a spectrum: controlled TE expression can contribute cis‐regulatory modules, non‐coding RNAs, chromatin states, and immune enhancers, whereas excessive or misregulated TE activity can promote genome instability, innate immune activation, and inflammatory pathology. To reduce conceptual redundancy, we organize these processes within an integrative framework, the Transposon Circuitry Theory of Immune Regulation (TACTIR), which links TE‐derived modules, chromatin control of TE transcription, stress pathways, and immune gene networks. In this model, TE‐derived enhancers and transcription factor motifs may shape stimulus‐responsive gene expression, while TE transcripts and reverse‐transcribed products can engage innate immune sensors under specific states of derepression. We also discuss how TE‐linked regulatory states may contribute to trained immunity and T‐cell dysfunction, while distinguishing established mechanisms from inferred or hypothetical links. Clinically, we place TE‐mediated insertional disease, cancer‐associated TE dysregulation, and immune modulation in appropriate context, noting that TE insertions account for a minority of genetic disease cases but provide important mechanistic examples of genome vulnerability. Finally, we consider how genome‐editing approaches and transposon‐derived tools can clarify TE function and enable translational applications. Together, this Review frames TEs as regulated genomic elements that connect genome plasticity, immune responsiveness, and disease susceptibility.

## Summary

- Transposable elements constitute nearly half of the human genome and function as context‐dependent regulators of genome organization, transcription, and immune responses.
- This Review introduces the Transposon Circuitry Theory of Immune Regulation (TACTIR), an integrative framework connecting TE‐derived regulatory modules, chromatin control, cellular stress pathways, and immune gene networks.
- Controlled TE activity can contribute cis‐regulatory elements, non‐coding RNAs, chromatin states, and stimulus‐responsive immune enhancers, whereas excessive derepression can promote genome instability and inflammatory signaling.
- Under permissive cellular conditions, TE‐derived RNA and reverse‐transcribed products can engage innate immune pathways, including RIG‐I/MDA5‐MAVS and cGAS‐STING signaling.
- TE‐associated regulatory states may influence cancer biology and immune‐cell function; however, experimentally established mechanisms must be distinguished from inferred or hypothetical associations.

## 1 Introduction

The conceptual framework for understanding transposable elements (TEs) was first established by Barbara McClintock in 1953, through her seminal work in *Zea mays*. McClintock identified mobile genetic elements capable of altering their position within the genome, specifically describing the Dissociation (Ds) element, which can interrupt gene expression upon insertion, and the Activator (Ac) element, which governs the timing of such transposition events. Her observations provided a mechanistic explanation for variegated kernel pigmentation and, more broadly, introduced the paradigm that endogenous mobile DNA sequences can generate heritable genomic variation [1]. This pioneering research laid the conceptual groundwork for later models describing transposon‐centered molecular complexes, often referred to as the transposome, comprising the mobile element, its encoded enzymatic machinery, and the cis‐acting sequences required for mobilization, thereby underscoring the dynamic and regulatory roles of TEs in genome architecture and evolution.

Contemporary genomic analyses have confirmed that TEs constitute a substantial fraction of eukaryotic genomes. They are generally classified into two major categories: Class I elements, or retrotransposons, which transpose via an RNA intermediate through a copy‐and‐paste mechanism; and Class II elements, or DNA transposons, which mobilize using a DNA intermediate through cut‐and‐paste or replicative DNA‐based mechanisms [2]. In mammalian genomes, TEs represent approximately 50% of the total DNA content. These elements often harbor sequences capable of recruiting the host's transcriptional machinery, enabling regulated transcription of transposon‐derived RNAs and, in rare cases, facilitating retrotransposition. Importantly, even when transposition activity ceases, TE‐embedded regulatory sequences can persist and exert long‐term effects on host gene expression [3]. Recent advances in genome sequencing and editing technologies have enabled systematic investigation of these regulatory functions, revealing the profound impact of TEs on gene regulation and genome evolution.

## 2 Structural Classification and Autonomous Potential of Human Retrotransposons

Retrotransposons, a subclass of Class I transposable elements, can be further categorized into two groups: those possessing long terminal repeats (LTRs) flanking their sequence, and those lacking them, referred to as non‐long terminal repeat (non‐LTR) retrotransposons. TE families are subsequently subclassified based on the presence of different types of flanking repeats and protein coding sequences [4]. When these proteins are present and functional, TEs are considered autonomous, as they encode the protein machinery required to mediate their own mobilization. Conversely, if these proteins are non‐functional or absent, the element is termed non‐autonomous; although still capable of transposition, its mobility is dependent on the machinery encoded by an autonomous TE [5].

In the human genome, detectable ongoing or recent retrotranspositional activity is restricted to three non‐LTR retrotransposon families: Alu, long interspersed nuclear element‐1 (LINE‐1; L1), and SINE‐R/VNTR/Alu (SVA) [6]. Among these, L1 is the sole retrotransposon with autonomous mobilization capability, while Alu and SVA depend on the protein machinery encoded by L1 for their retrotransposition. A full length L1 element is approximately 6 kilobases (kb) in size and contains two open reading frames (ORFs), which encode the protein machinery necessary for its mobilization and integration. L1 represents the most prevalent TE type in the human genome in terms of its contribution to the total number of bases, constituting approximately 17% of its sequence [7].

### 2.1 Mutagenesis Driven by Transposable Elements and Mechanisms Preserving Genome Integrity

Transposable elements (TEs) are mobile DNA sequences that can change their position within the genome, often by duplication. This mobility, known as transposition, can have mutagenic and reorganizational effects on the genome [8]. One of the earliest documented cases linking TEs to human disease involved patients with hemophilia A, in whom de novo L1 retrotransposon insertions disrupted the F8 gene encoding coagulation factor VIII.These insertions, absent from the parental genomes, provided direct evidence that retrotransposition can occur spontaneously in the human germline and act as an active mutational force in humans. L1 elements, belonging to Class I transposable elements, propagate via a “copy‐and‐paste” mechanism involving an RNA intermediate and reverse transcription. The L1‐encoded open reading frame 2 protein (ORF2p) is required for LINE‐1 retrotransposition, with the detailed target‐primed reverse transcription mechanism described below, enabling the element to cleave genomic DNA and integrate a new complementary DNA (cDNA) copy at a different site [9]. More recent genome‐wide sequencing studies have expanded this early observation by showing that Alu, LINE‐1 and SVA elements remain actively mobile in human genomes, generating new mobile element insertions in germline and, in some contexts, somatic tissues; these insertions can disrupt exons or other functional genomic regions and are increasingly detectable through whole‐genome and long‐read sequencing approaches [9, 10]. Together, the classical hemophilia A case and contemporary sequencing‐based analyses highlight the clinical relevance of endogenous mobile elements as ongoing contributors to human genetic variation, genome instability and disease susceptibility.

In the face of the constant threat posed by TEs, genomes have evolved defense mechanisms across eukaryotes. In plants, one of the best characterized TE silencing mechanisms is RNA‐directed DNA methylation (RdDM), which constrains transposon proliferation through small RNA‐guided DNA methylation [11, 12]. In mammals, analogous genome‐protective functions are mediated primarily through DNA methylation, histone H3K9 trimethylation, and small RNA‐associated pathways [13]. At the chromatin level, SET domain bifurcated histone lysine methyltransferase 1 (SETDB1)‐mediated deposition of histone H3 lysine 9 trimethylation (H3K9me3) represents a central repressive mechanism for retroelements, acting in cooperation with KRAB‐associated protein 1 (KAP1; also known as TRIM28)‐associated pathways to promote heterochromatin formation and transcriptional silencing of LINE‐1 and endogenous retroviral families [14]. This repression is further reinforced by human silencing hub (HUSH)‐mediated surveillance systems, which couple transcribed retroelements and LINE‐1‐containing loci to chromatin‐repressive machinery, including SETDB1‐dependent H3K9me3 deposition, thereby limiting young LINE‐1 activity in human cells [15, 16]. In the germline, PIWI‐interacting RNA (piRNA)‐associated pathways provide an additional layer of defense by guiding retrotransposon recognition and supporting de novo DNA methylation, protecting the heritable genome during stages of epigenetic reprogramming [17]. Together, these multilayered systems restrict retrotransposon expression and insertional mutagenesis, preserving genome integrity while allowing some TE‐derived sequences to be retained as regulatory substrates in mammalian genomes [3, 15, 16].

Despite these silencing mechanisms, a fundamental question in genomic biology is how TEs persist in genomes, given their frequently deleterious properties. Purifying selection acts to eliminate TEs that confer a high biological cost to the organism [18], effectively removing variants that reduce individual fitness and maintaining the integrity of functional sequences. However, elements with mildly deleterious, neutral, or context‐dependent effects often escape eradication. This is supported by population‐scale genome sequencing studies, in which 36,699 non‐reference mobile element insertions were identified across 5,675 human genomes, demonstrating that Alu, LINE‐1, and SVA insertions persist as widespread structural variants in modern populations. Although coding insertions show clear evidence of selective constraint, mobile element insertions are estimated to account for approximately 9.3% of protein‐truncating events per genome, underscoring their continuing mutagenic potential [19]. Crucially, persistence does not necessarily imply neutrality: Genotype‐Tissue Expression (GTEx)‐based analyses identified 20,545 polymorphic mobile element insertions across 639 individuals and linked them to tissue‐specific regulatory effects, including 3,522 expression quantitative trait loci (eQTLs) and 3,717 splicing quantitative trait loci (sQTLs) [20]. These findings suggest that TE persistence reflects a dynamic balance between purifying selection, host repression, genomic context, and regulatory impact, allowing some insertions to be eliminated, others to remain as low‐frequency variation, and a subset to actively shape human gene expression and transcript diversity.

The persistence of even sparingly deleterious TEs, particularly autonomous retrotransposons, represents a constant risk. In humans, this risk is concentrated in a limited subset of retrotransposition‐competent L1 source elements rather than in the large majority of degenerated TE copies. More than 99.9% of L1‐derived sequences contain 5′ truncations, internal rearrangements, deletions or point mutations that render them unable to retrotranspose, but an average human genome still contains approximately 80‐100 active full‐length human‐specific L1s, with a small subset of highly active “hot‐L1” elements accounting for much of the remaining retrotransposition activity [21]. Importantly, retrotransposition competence does not necessarily imply constitutive expression: only a select few L1 loci are expressed in any given cell line or tissue, and their transcription depends on a permissive epigenetic context that includes open chromatin, activating histone modifications, promoter hypomethylation and enhancer interactions. These findings suggest that burst‐like increases in L1 activity are most likely to emerge when epigenetic repression is locally weakened, such as during tumor‐associated or aging‐associated epigenetic dysregulation, allowing specific source elements to generate new insertions and, indirectly, to support mobilization of non‐autonomous elements such as Alu [21, 22]. The genomic consequences of this relaxation are starkly visible in disease: pan‐cancer whole‐genome analyses of 2,954 tumors identified 19,166 somatically acquired retrotransposition events affecting 35% of samples, demonstrating that L1 activation can reshape cancer genomes through insertions, 3′ transductions, deletions, translocations, and large‐scale duplications [23]. Importantly, somatic L1 activity is not restricted to malignant cells; single‐cell clone sequencing from normal tissues identified 1,708 somatic L1 retrotransposition events enriched in colorectal epithelium. These events correlated with age and arose from 34 retrotransposition‐competent L1 source elements, with lower‐frequency source alleles exhibiting higher activity [23, 24]. Long‐read sequencing has further refined this landscape, revealing 1,495 somatic insertions in colorectal cancers and distinct structural differences between somatic and germline retrotransposition events [25]. Together, these findings indicate that a minor pool of active source elements continually replenishes the genomic repertoire of TE insertions. Conversely, horizontal transfer (HT) must be framed cautiously in human‐centered biology. While HT drives genome evolution across distant taxa, a comprehensive search across 27,949,823 retrotransposons from humans and domestic mammals found no plausible evidence of recent horizontal transfer [26]. This underscores that contemporary human TE dynamics are strictly governed by vertical inheritance, endogenous source‐element activity, and context‐dependent derepression.

The functional impact of transposons primarily manifests through their ability to reshape the genome and modulate gene expression via direct interactions with host DNA and transcriptional machinery. For instance, transposon insertions can introduce promoters, enhancers, or transcription factor binding sites thereby altering chromatin accessibility and the transcription initiation rate of nearby genes [27]. In addition to insertional effects, LINE‐1‐mediated retrotransposition can mobilize adjacent host genomic sequences through 3′ transduction, a process in which transcription extends beyond the LINE‐1 polyadenylation signal and incorporates downstream flanking DNA into a new insertion. Depending on the genomic context of the source element, these transduced sequences may include exonic, intronic or regulatory regions, potentially generating chimeric transcripts, altering splicing patterns or modifying gene structure [28]. Transposon activity can also influence genome stability by creating DNA double‐strand breaks or generating recombinogenic substrates, contributing to large‐scale chromosomal rearrangements such as deletions, duplications and inversions [29].

Molecular mechanisms of transposition differ fundamentally between DNA transposons and retrotransposons. DNA transposons are generally mobilized by transposase proteins encoded by autonomous elements, and these proteins can also act in trans to mobilize non‐autonomous copies. These enzymes typically contain catalytic DDE/D motifs that coordinate DNA cleavage and strand transfer, recognize terminal inverted repeats at the element boundaries, catalyze excision from the donor locus, and integrate the element into a new genomic site. Host DNA repair machinery then resolves the insertion intermediate, often generating short direct target site duplications (TSDs) at the insertion site, a molecular hallmark of DNA transposon mobilization [30]. Conversely, retrotransposons employ a “copy‐and‐paste” mechanism involving an RNA intermediate. They are first transcribed into RNA, which then serves as a template for reverse transcription. In LTR retrotransposons, integration is mediated by a dedicated integrase, whereas non‐LTR retrotransposons such as LINE‐1 utilize Target‐Primed Reverse Transcription (TPRT). During TPRT, the element‐encoded ORF2p protein nicks genomic DNA and uses its reverse transcriptase activity to synthesize cDNA directly at the target site. This process generates characteristic hallmarks of LINE‐1‐mediated retrotransposition, including TSDs, poly(A) tails, and frequent 5′ truncations [31].

Regulation of transposable elements is a complex and multifaceted process involving layered epigenetic and genetic silencing pathways that preserve genomic integrity. DNA methylation at TE promoters and CpG‐rich regulatory regions serves as a primary mechanism to promote compact chromatin states and suppress transcription [32]. This is tightly coordinated with repressive histone modifications [33]. In parallel, sequence‐guided repression operates through divergent somatic and germline systems. In the human germline, Piwi‐interacting RNAs (piRNAs) provide critical defense by guiding transcript degradation, *de novo* DNA methylation, and chromatin silencing, whereas in somatic tissues, TE control is largely driven by Krüppel‐associated box zinc finger proteins (KZFPs) collaborating with the KAP1 corepressor complex [34]. Disruption of these homeostatic mechanisms leads to transposon derepression. The resulting accumulation of TE transcripts and proteins not only drives genomic instability but can also trigger aberrant innate immune responses, ultimately contributing to pathologies such as oncogenesis and neurodegenerative disorders.

### 2.2 Functional Testing of Transposable Elements and CRISPR‐Directed Integration Systems

Methodologies to study transposable elements have diversified to assess not only their genomic annotation but also their regulatory state, transcriptional output and potential mobility. Contemporary bioinformatic and machine‐learning approaches combine repeat annotation with sequencing‐based datasets, including TE‐derived transcript profiles, small RNA data, DNA methylation and other epigenetic features, to infer locus‐specific regulation and silencing of mobile elements [35]. However, the presence of TE sequences alone does not necessarily indicate retrotranspositional activity. Functional assessment must therefore consider whether key steps of the retrotransposon life cycle are engaged, including transcriptional reactivation, RNA accumulation, ribonucleoprotein formation, reverse transcription into cDNA and, when detectable, *de novo* genomic integration. Mapping these molecular layers is crucial for distinguishing inactive TE remnants from transcriptionally active or mobilization‐competent elements, and for understanding how retrotransposon dysregulation can contribute to insertional mutagenesis, genome instability, cellular stress responses and innate immune sensing [36].

**Figure 1:**
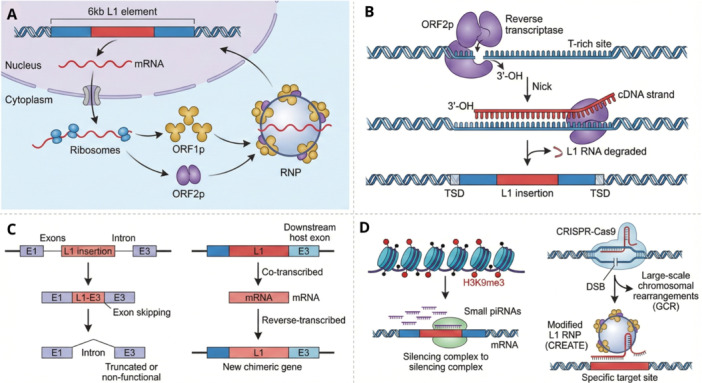
Cellular framework of TACTIR linking transposable element‐derived regulatory DNA, nucleic‐acid sensing, and stress‐associated feedback. Figure 1 Legend. The Transposon Circuitry Theory of Immune Regulation (TACTIR) proposes that transposable elements (TEs) influence immune regulation through interconnected but mechanistically distinct routes. (A) TE‐derived regulatory sequences can participate in enhancer‐promoter communication, chromatin regulation, transcriptional control, RNA processing, and host gene regulation, while TE‐derived RNA or cDNA may engage innate immune sensors under permissive cellular states. (B) TE activity can be viewed as a continuum, ranging from low or moderate regulatory output to excessive TE derepression associated with retroelement‐derived RNA/cDNA accumulation, genome instability, and innate immune activation. (C) TE‐linked chromatin organization may contribute to cellular plasticity, enhancer rewiring, and age‐ or stress‐associated inflammatory states. (D) Organelle stress, mitochondrial signals, ER‐Golgi trafficking, and endolysosomal pathways can feed back onto TE expression and TE‐derived nucleic‐acid sensing, linking TE derepression to RIG‐I/MDA5, cGAS‐STING, inflammation, senescence, and stress‐adaptive programs. Together, these mechanisms illustrate TACTIR as a compartmentalized framework rather than a single linear pathway.

The functional impact of transposon activity is another key aspect of testing. Transposon insertions can have profound consequences, ranging from direct gene disruption and altered gene expression to the induction of large‐scale chromosomal rearrangements [37]. For instance, targeting highly repetitive human endogenous retrotransposons like LINE‐1 and Alu with clustered regularly interspaced short palindromic repeats (CRISPR)‐associated protein 9 (Cas9), commonly referred to as CRISPR‐Cas9, in human cells has been shown to induce global chromosome rearrangement (GCR). This leads to a large number of DNA double‐strand breaks (DSBs) and results in numerous inversions, translocations, and copy number variations (CNVs). These genomic alterations, in turn, profoundly reshape the epigenetic and transcriptomic landscapes of the cells, with significant changes in gene expression related to pathways like p53, DNA repair, and cell cycle regulation [38]. Testing at this level includes RNA sequencing (RNA‐seq) techniques to evaluate changes in gene expression profiles after transposition events, and chromatin assays such as assay for transposase‐accessible chromatin sequencing (ATAC‐seq) or chromatin immunoprecipitation sequencing (ChIP‐seq) to analyze modifications in chromatin accessibility or transcription factor binding induced by transposon insertion [39]. Characterizing these modifications is vital for linking transposon activity to specific phenotypes or disease states, as observed in certain cancers and neurodevelopmental disorders. Figure 1.

Testing and studying transposons are primarily indicated in research and advanced diagnostic contexts, not as routine clinical tests for patients. However, they are crucial in the following scenarios.

### 2.3 Genetic Disease Research

When there is clinical, genomic or experimental suspicion that TE activity contributes to disease through insertional mutagenesis, altered gene structure, aberrant splicing, ectopic promoter or enhancer activity, or TE derepression. In cancer, somatic LINE‐1 activity has been demonstrated by whole‐genome analyses to generate insertions, 3′ transductions, deletions, translocations and other structural rearrangements that can remodel tumor genomes. In neurological and neurodegenerative disorders, the evidence is more heterogeneous and should be framed cautiously: L1 expression, copy number changes, intragenic L1 insertions, heterochromatin relaxation, *TAR* DNA‐binding protein 43 (TDP‐43)/tau‐associated TE dysregulation, DNA damage and neuroinflammatory pathways have been reported or proposed in patient tissues and disease models, but their causal contribution remains context‐dependent and incompletely resolved [40, 41, 42].

### 2.4 Genome Instability Studies

In diseases or cellular states characterized by high mutational burden, chromosomal rearrangements or unexplained structural variation, TEs should be evaluated as potential sources or amplifiers of genome instability. Mechanistically, LINE‐1 ORF2p endonuclease and reverse transcriptase activities can generate new insertions, while L1‐mediated 3′ transduction, insertion‐associated deletions, recombination between repetitive copies, and breakage‐fusion‐bridge cycles can produce copy‐number alterations, interchromosomal translocations and broader genome remodeling [23, 43].

### 2.5 Personalized Medicine and Therapy Development

In oncology, TE profiling may help stratify tumors by retrotransposition burden, epigenetic derepression, TE‐derived RNA/protein output, immune visibility and therapeutic vulnerability. This is not yet a universal clinical tool, but recent cancer studies support its translational relevance: TE reactivation can produce double‐stranded RNA (dsRNA) and viral mimicry responses, TE‐derived or TE‐chimeric peptides may contribute to tumor antigen presentation, and excessive L1 activity can impose DNA damage or replication stress [43, 44]. In high‐grade serous ovarian cancer, de novo L1 insertions were shown to be dynamic in vivo and to contribute to intrapatient genomic heterogeneity, supporting the idea that L1 burden and L1 profiles may inform tumor evolution and potential vulnerabilities rather than simply serving as passive markers [43, 45].

### 2.6 Developmental Biology

For embryonic development and cell differentiation, TE analysis should distinguish regulated TE transcription from active mobilization. In this context, TE RNAs and TE‐derived regulatory sequences are studied as components of developmental gene regulation, chromatin remodeling and cell‐state transitions, whereas uncontrolled retrotransposition is not assumed unless direct insertional evidence is available [42, 46].

CRISPR (Clustered Regularly Interspaced Short Palindromic Repeats) is a groundbreaking gene editing technology that has transformed scientists' ability to precisely modify DNA. Originally discovered as a bacterial immune system defending against viruses, CRISPR has been adapted to allow researchers to cut and edit specific DNA sequences in almost any organism [47]. The system typically consists of two key components: a Cas enzyme (like Cas9, acting as “molecular scissors”) and a guide RNA (gRNA) that directs the Cas enzyme to the target DNA sequence. Once Cas binds to the complementary DNA sequence, it makes a cut, and the cell's natural repair machinery then steps in to correct or modify the gene, which can result in the deletion, insertion, or correction of genetic sequences. This editing capability is rooted, at a molecular level, in the induction of a double‐strand break (DSB) in DNA at a specific genomic site. Once the DSB occurs, the cell activates its own DNA repair systems: non‐homologous end joining (NHEJ) and homology‐directed repair (HDR) [48, 49]. NHEJ is an error‐prone mechanism that often introduces small insertions or deletions (indels) at the cut site, potentially leading to gene inactivation. In contrast, HDR is a high fidelity repair mechanism that uses a homologous DNA template to precisely repair the break, allowing for the insertion of specific genetic sequences or the correction of mutations [50, 51]. Recent studies have shown that engineered Cas9 nucleases (vCas9) can be designed to alter the commitment of these repair pathways, suppressing NHEJ and favoring HDR or microhomology‐mediated end joining (MMEJ). This strategy has been shown to bias repair outcomes toward more predictable and precise editing in experimental systems, a crucial aspect for therapeutic applications where accuracy is paramount and off‐target mutations must be minimized [52].

The relationship between CRISPR and transposons has evolved from CRISPR's ability to degrade mobile genetic elements (as part of the bacterial immune system) to the co‐option of CRISPR/Cas systems by certain transposons for programmable DNA integration. A notable recent development is CRISPR‐enabled autonomous transposable element (CREATE), which combines the precision of CRISPR/Cas9 with the RNA‐mediated gene insertion capability of human LINE‐1 (L1) retrotransposons [53]. This experimental system enables programmable insertion of kilobase‐scale genetic payloads into defined genomic loci without requiring donor DNA templates or intentionally induced DNA double‐strand breaks, relying instead on RNA intermediates derived from the L1 retrotransposition cycle. Although still preclinical, CREATE provides a proof of concept for RNA‐based targeted gene insertion and may inform future strategies for safer and more precise gene delivery. CRISPR‐associated transposons (CASTs) are natural complexes that combine the CRISPR‐Cas machinery with Tn7‐like transposons [53, 54]. These systems enable programmable DNA integration through a mechanism known as RNA guided transposition, where the guide RNA not only directs the Cas enzyme to the target site but also recruits the transposon machinery for insertion [55].

### 2.7 Toward a Unified Model of Transposon‐Mediated Genome Regulation

Retrotransposons such as LINE‐1, Alu and SVA can contribute promoter‐like and enhancer‐like sequences that become incorporated into host gene‐control landscapes, functioning in specific cellular contexts as active genomic regulatory modules rather than passive remnants. This has been particularly described in human normal and malignant haematopoiesis, where TE sequences can reshape transcriptional programs, create enhancers or promoters, and influence cellular identity [56]. Genome‐wide chromatin interaction analyses in human K562 cells support the enhancer‐promoter looping model and identify EP300 and SWI/SNF‐associated chromatin remodeling as candidate contributors to promoter‐centered regulatory contacts [56, 57]. Complementary genome‐wide studies further show that 3D chromatin architecture, bromodomain‐containing protein 4 (BRD4), Mediator and cohesin regulate transcriptional bursting and gene‐network behavior, providing a mechanism by which TE sequences may influence transcription when they acquire active chromatin states [58]. More broadly, experimental work in splicing factor 3b subunit 1 (SF3B1)‐mutant cancer models demonstrates that defects in RNA processing and RNA polymerase II elongation can remodel chromatin landscapes, supporting a functional connection between transcriptional elongation, RNA processing and chromatin regulation [59].

In parallel, retrotransposon‐derived nucleic acids can become immunostimulatory under defined pathological or experimentally perturbed conditions. In human cellular models of Aicardi‐Goutières syndrome and adenosine deaminase acting on RNA 1 (ADAR1), Alu‐derived duplex RNAs have been experimentally shown to activate melanoma differentiation‐associated protein 5 (MDA5)‐mediated inflammatory signaling [60]. In aging and sirtuin 6 (SIRT6)‐deficient mouse models, LINE‐1 derepression leads to cytoplasmic LINE‐1 cDNA accumulation and activation of type I interferon responses through cyclic GMP‐AMP synthase (cGAS)‐dependent pathways [60, 61]. Similarly, in senescent human and mouse cells, LINE‐1 activation has been linked to cytoplasmic L1 cDNA, interferon signaling and age‐associated inflammation [62]. Together, these findings provide a mechanistic foundation for viewing retrotransposons as context‐dependent regulatory elements that influence transcriptional plasticity, chromatin regulation and innate immune signaling, while emphasizing that immune activation reflects specific states of derepression, nucleic acid accumulation or impaired self‐tolerance rather than constitutive activity of all retrotransposon transcripts.

Accordingly, the effects of retroelement‐derived transcripts should be interpreted as context‐dependent rather than as a consequence of genomic presence alone. Genome‐wide enhancer‐promoter RNA interaction analyses have shown that Alu‐containing RNAs can contribute to enhancer‐promoter selectivity through complementary RNA‐RNA interactions, supporting a regulatory role for Alu‐derived transcripts in transcriptional organization [63]. This regulatory activity should be distinguished from pathological retrotransposon derepression, in which failure of epigenetic repression or nucleic acid surveillance allows retroelement‐derived RNAs or reverse‐transcription products to accumulate. For example, HUSH‐mediated silencing recognizes intronless reverse‐transcribed elements and represses transcriptionally active LINE‐1 loci. In this context, the protective role of HUSH refers to its ability to limit the immediate deleterious effects of newly integrated or transcriptionally active retroelements, rather than to a single measurable cutoff of retroelement transcription [64]. Experimentally, HUSH depletion in human cell lines and primary fibroblasts induces interferon‐stimulated genes through MDA5‐ and retinoic acid‐inducible gene I (RIG‐I)‐dependent sensing of double‐stranded RNAs (dsRNAs), coinciding with the derepression of primate‐conserved and hominid‐specific LINE‐1 elements [65]. These senescence‐associated findings are consistent with cGAS‐STING‐linked type I interferon activation [62, 66]. Taken together, these findings support an integrative model in which TE RNAs, LINE‐1 derepression, cytosolic nucleic acid accumulation, and innate immune activation may converge under specific cellular conditions. However, current evidence does not establish a single linear pathway in which LINE‐1 endonuclease activity directly causes micronuclei formation, dsRNA release, and the simultaneous activation of cGAS‐STING and RIG‐I/MDA5.

Consistent with this context‐dependent regulation, each cell type maintains a characteristic epigenetic state at retroelement loci that limits TE transcription, chromatin accessibility, and, where relevant, retrotransposition potential. Rather than representing a fixed quantitative cutoff, this state reflects a dynamic balance between repressive chromatin marks, DNA methylation, transcription factor availability, and developmental or inflammatory cues. Mechanistically, KAP1 contributes to KZFP‐mediated repression, while its HP1 box promotes HP1α‐dependent chromatin compaction. In mouse embryonic stem cells, structural, biochemical, and chromatin profiling experiments show that the KAP1–HP1α interaction is required to maintain inaccessible chromatin at endogenous retroviral elements [67]. DNA methylation and other DNA modifications further reinforce TE repression across mammalian genomes, with *de novo* and maintenance methylation contributing to the long‐term silencing of retrotransposon‐derived sequences [68]. Together, these epigenetic layers reduce inappropriate retroelement transcription and help preserve cell‐type‐specific transcriptional identity.

This context‐dependent behavior is also reflected across developmental and aging‐associated regulatory states. Pluripotent cells operate within highly plastic regulatory enhancer landscapes characterized by enhancer remodeling and dynamic promoter‐enhancer communication. In human naïve and primed pluripotent stem cell states, OCT4, SOX2 and NANOG undergo extensive redistribution across state‐specific enhancer classes, accompanied by changes in enhancer activity, enhancer‐promoter interactivity and target gene expression [69]. These findings indicate that developmental plasticity is supported by flexible enhancer‐promoter relationships rather than fixed configurations. As cells progress toward differentiated states, regulatory landscapes become increasingly lineage‐specific: human regulatory DNA maps show that DNase I‐hypersensitive site patterns encode cell fate, lineage relationships and developmental maturity, while multi‐omic profiling of T helper cell differentiation shows that naïve CD4 + T cells acquire cell‐type‐specific transcriptional programs, chromatin accessibility profiles and enhancer‐promoter loops during differentiation into Th1 and Th17 states [70, 71]. During aging, however, enhancer‐promoter organization can become destabilized. In murine muscle stem cells, low‐input multi‐omic profiling has shown that aging is associated with widespread enhancer activation, extensive three‐dimensional genome rewiring, loss of myogenic identity and sustained inflammatory signaling; in this model, proinflammatory pathways correlate with endogenous retroviral expression and NFκB activity [72].

Cell‐state‐dependent regulation of retroelements may also intersect with organelle stress and quality‐control pathways, but the available evidence supports this relationship only in specific contexts rather than as a single established linear mechanism. Under homeostatic conditions, retroelement transcription, RNA/cDNA persistence and retrotransposition potential are limited by epigenetic repression, post‐transcriptional surveillance and degradation pathways. Direct TE‐organelle connections have been demonstrated in selected systems. In human dopaminergic LUHMES cells, pharmacological inhibition of mitochondrial complex I increases LINE‐1 open reading frame 1 protein (ORF1p) expression in a reactive oxygen species (ROS)‐sensitive manner, with supporting observations in treated mouse midbrain tissue [73]. In human regulatory T cells from autoimmune liver disease, HERV1‐env expression induces ER stress and activates unfolded protein response (UPR) branches involving inositol‐requiring enzyme 1 (IRE1), activating transcription factor 6 (ATF6) and protein kinase RNA‐like ER kinase (PERK), linking an endogenous retroviral protein to immune‐cell dysfunction through UPR signaling [73, 74]. A separate organelle‐associated restriction mechanism has also been described for LINE‐1: STING can bind LINE‐1 ORF1p and promote its trafficking through ER‐Golgi/endolysosomal compartments to Rab7‐positive lysosomes for degradation, thereby restricting LINE‐1 retrotransposition independently of cGAS and interferon induction [75]. These TE‐specific examples should be distinguished from broader organelle stress pathways. ER stress and lysosomal dysfunction under oxidative stress are well‐established features of cancer cell adaptation and proteostasis failure, but direct retrotransposon involvement is not central to those mechanisms unless demonstrated in the specific model [76]. Similarly, mitochondrial RNA leakage during senescence activates RIG‐I/MDA5‐mitochondrial antiviral‐signaling protein (MAVS) signaling and contributes to the senescence‐associated secretory phenotype (SASP), but this reflects mitochondrial nucleic acid sensing rather than direct TE‐derived RNA sensing [76, 77]. Broader evidence also indicates that retrotransposon RNA and cDNA intermediates can be regulated by host degradation and surveillance pathways [31]. However, current evidence does not establish a universal self‐reinforcing loop in which mitochondrial, ER or lysosomal stress directly drives TE activation and TE‐derived nucleic acids act as the primary mediators of all downstream inflammatory responses. Future research will be required to determine whether organelle‐TE interactions represent a broadly conserved stress‐response mechanism or instead reflect highly cell‐type‐specific adaptations.

After separating TE nucleic acid sensing from organelle stress pathways and TE cis‐regulatory functions, we propose the Transposon Circuitry Theory of Immune Regulation (TACTIR) as a unifying model for how TEs can shape immune gene regulation. In this model, TEs do not function primarily as autonomous immune sensors; rather, TE insertions can provide lineage‐ and cell‐type‐specific enhancers or promoters that can be incorporated into cytokine‐ and pathogen‐responsive transcriptional circuits. A clear experimental anchor for this cis‐regulatory logic is provided by lineage‐specific MER41 endogenous retroviruses (ERVs), whose LTRs dispersed IFN‐inducible STAT1‐responsive enhancer sequences across mammalian genomes. In human cells, MER41.AIM2 gains enhancer‐associated histone H3 lysine 27 acetylation (H3K27ac) after IFNγ stimulation, and CRISPR‐Cas9 deletion of this element abolishes IFNγ‐induced AIM2 expression and reduces infection‐induced caspase‐1 activation, linking an ERV‐derived enhancer to AIM2‐dependent inflammasome function [78]. LINE‐derived regulatory elements further support this model: an intronic L1M2a element within interferon alpha and beta receptor subunit 1 (IFNAR1) carries enhancer‐associated chromatin features despite HUSH‐linked H3K9me3 repression, and CRISPR deletion of its enhancer region reduced basal IFNAR1 expression, abolished IFNβ‐induced IFNAR1 upregulation, and dampened downstream type I interferon responses in B lymphoblastoid cells [79]. TACTIR is proposed to extend this logic beyond the IFN‐STAT axis by considering how TE sequences may contribute to inflammatory enhancer evolution. Comparative epigenomic analyses indicate that primate‐specific ERVs and Alu elements introduced functional NF‐κB and interferon regulatory factor 1 (IRF1) motifs into immune‐cell enhancers, with evidence that some Alu‐derived NF‐κB sites underwent positive selection and increased predicted binding affinity in humans, suggesting evolutionary tuning of inflammatory responsiveness [80]. In infection‐responsive human macrophages, TE families such as MER44, THE1, Tigger3 and MLT1 are enriched within stimulus‐induced accessible chromatin near differentially expressed genes and frequently contain activator protein 1(AP‐1) and NF‐κB motifs, consistent with a role for TE sequences in inducible innate immune programs [81]. Finally, single‐cell multiomic analyses of human peripheral blood immune cells show that TE‐overlapping enhancers are highly cell‐type‐specific, with monocytes displaying particularly strong enrichment; more than 30% of epigenetically annotated monocyte enhancers overlap TE‐derived sequences and are frequently associated with myeloid regulators such as PU.1, ATF4 and CEBP proteins [81, 82].

Building on the distinction between stress‐dependent transposon regulation, organelle‐associated immune signaling and cis‐regulatory TE activity, this framework suggests that selected TE‐derived modules may contribute to immune memory‐like states by shaping the enhancer landscape through which canonical immune signaling pathways operate. Rather than acting as independent immune sensors, selected ERV, LINE and SINE/Alu‐derived sequences can function as lineage‐ and cell‐state‐dependent regulatory substrates embedded within immune enhancer architectures.

Importantly, this contribution may involve not only the presence of TE‐derived motifs, but also their position within enhancer domains. In CD8 + T lymphocytes, TE subfamilies show a non‐random distribution across enhancer architecture: ERV‐derived sequences are enriched in accessible enhancer cores and carry immune‐related transcription factor motifs, whereas SINE‐ and LINE‐derived sequences are enriched in enhancer boundary regions associated with histone H3 lysine 4 monomethylation (H3K4me1)‐marked chromatin [83]. During inflammatory stimulation, these regulatory architectures require chromatin remodeling to become functionally engaged. In macrophages, SWI/SNF chromatin remodeling complexes cooperate with lineage‐determining and stimulus‐responsive transcription factors, including PU.1, AP‐1, NF‐κB and interferon‐responsive factors, to regulate stimulus‐induced chromatin accessibility, H3K27ac dynamics, enhancer transcription and inflammatory or interferon‐stimulated gene activation [84]. More generally, enhancer‐associated chromatin states involve features such as H3K4me1, H3K27ac, CBP/p300 activity and RNA polymerase II‐associated transcription, but these features should be interpreted as enhancer biology rather than as evidence that every TE‐derived locus acquires all marks in every immune context [85, 86]. Consequently, TE regulatory activity is neither uniformly derepressed nor uniformly silent. Instead, endogenous retroelement expression and regulatory potential are heterogeneous across human somatic tissues and cell types, supporting a context‐dependent view in which selected TE‐derived loci may contribute to immune plasticity under defined cellular states [87]. A locus‐specific example of this context‐dependent regulatory complexity is provided by the human MHC class II region. In peripheral blood transcriptomes, SVA polymorphisms have been associated with coordinated transcription of clustered LINE, SINE/Alu, LTR/ERV and DNA transposon loci near HLA class II genes, suggesting that TE‐derived transcripts may participate in local regulatory variation within immune gene‐dense regions [88].

A more granular example comes from the internal organization of T cell enhancers. In mouse CD8 + T cells, integrative analyses of ATAC‐seq, histone modifications and transcriptomic changes showed that specific TE subfamilies are non‐randomly distributed across enhancer domains: ERV‐derived sequences, together with selected LINE and MIR elements, are enriched toward accessible enhancer cores and carry transcription factor recognition motifs relevant to T cell biology, whereas other SINE‐derived sequences are enriched in flanking or boundary regions associated with H3K4me1‐marked chromatin.83 Predicted enhancer‐gene assignments, supported in part by chromatin conformation data, further suggest that TE‐rich enhancers are linked to immune‐related processes such as leukocyte activation, lymphocyte differentiation and cytokine production, often overlapping functionally with TE‐poor enhancers rather than creating entirely novel regulatory programs [83]. Within TACTIR, these data are therefore best interpreted as evidence that TE‐derived sequences can contribute to cell‐state‐dependent enhancer use and regulatory robustness in T cells, while broader effects on immune memory, exhaustion or long‐term transcriptional persistence remain plausible but require more direct functional validation [83, 89].

At a broader evolutionary scale, TE‐derived inflammatory regulatory inputs are not restricted to lymphocyte enhancers or interferon receptor loci. Multi‐species analyses of tumor necrosis factor (TNF)‐stimulated primary aortic endothelial cells from human, mouse and cow identified multiple TE subfamilies associated with RELA/NF‐κB‐bound regions near TNF‐responsive genes, with several TE‐derived sites showing active enhancer features after inflammatory stimulation. In human cells, MER81 provides a strong experimentally tested example: RELA motifs within MER81 contributed to enhancer activity, and deletion of a MER81‐derived enhancer near IFNGR2 impaired TNF‐induced interferon gamma receptor 2 IFNGR2 expression. These findings support the broader principle that TE‐derived motifs can be incorporated into mammalian inflammatory regulatory networks, although this evidence should be framed as inflammatory cis‐regulatory evolution rather than direct proof of immune‐cell memory [90].

Together, the IFNAR1.L1M2a and MER81 examples suggest that selected TE‐derived elements can be inducibly engaged, but only within chromatin environments that permit controlled enhancer activation. At the molecular level, TACTIR can therefore be framed as a balance between epigenetic constraint and stimulus‐dependent regulatory engagement. The HUSH complex provides genome surveillance by repressing long, transcribed intronless mobile elements, including L1s and retroviral sequences, through transcription‐coupled H3K9me3‐associated silencing, thereby limiting widespread retroelement activity under homeostatic conditions [91].

However, this repression is not necessarily incompatible with local regulatory potential. This principle parallels broader immune enhancer biology, in which inflammatory stimulation can convert latent or poised regulatory regions into active enhancer states. Across mouse bone marrow‐derived and human monocyte‐derived macrophage systems, NF‐κB‐ and LPS‐associated inflammatory responses are linked to changes in H3K4me1, chromatin accessibility, H3K27ac, enhancer RNA signatures, and enhancer‐linked gene expression, with some enhancer‐associated states persisting after stimulus withdrawal [92, 93].

Building from the partially retained enhancer states described above, TACTIR next predicts that TE‐derived enhancer modules may influence how innate immune cells respond to later inflammatory stimulation. Here, “activation thresholds” should be understood as the degree of prior stimulation, chromatin accessibility and transcription factor engagement required for a macrophage or other innate immune cell to mount, attenuate or amplify a secondary response. Recent work provides a mechanistic foundation for this idea. Sequential inflammatory stimulation can induce macrophage memory at the single‐cell level by reshaping NF‐κB signaling dynamics and chromatin accessibility, producing priming or tolerance depending on stimulus identity, dose and duration [94]. Similarly, interferon signaling rapidly remodels macrophage chromatin accessibility and three‐dimensional genome organization at interferon‐stimulated gene loci, with ISGF3/STAT‐dependent regulation contributing to both homeostatic and induced accessibility states [95]. These studies indicate that innate immune responsiveness is not determined only by cytokine abundance or receptor activation, but also by the chromatin landscape available to inflammatory transcription factors.

Within TACTIR, TE‐derived enhancers should therefore be framed as candidate modulators of activation thresholds only when they are embedded within these stimulus‐responsive regulatory networks. This contribution is likely to be family‐ and locus‐specific: Alu‐, LINE‐, and ERV‐derived sequences may differ in the transcription factor motifs, chromatin features and local regulatory architectures they provide, rather than acting as a uniform TE class. This does not mean that TEs independently generate trained immunity. Rather, selected TE‐derived loci may alter the permissiveness, amplitude or persistence of innate immune transcriptional responses by contributing transcription factor motifs, enhancer‐like chromatin features or local regulatory architecture. This controlled cis‐regulatory role should remain distinct from pathological retrotransposon derepression, in which LINE‐1 RNA, cDNA or retrotransposition‐associated products can activate innate immune pathways and promote inflammation [96].

Beyond modulating the permissiveness or amplitude of innate immune responses, TACTIR also provides a way to consider how selected TE‐derived elements may contribute to the spatial organization and partial stabilization of stimulus‐dependent chromatin states. In macrophages, inflammatory activation is accompanied by coordinated temporal changes in chromatin accessibility, enhancer H3K27ac, three‐dimensional chromatin contacts and gene expression, with looped enhancer‐promoter pairs showing ordered relationships between enhancer acetylation and transcriptional induction during activation [97]. These data support the broader principle that immune stimulation is interpreted through dynamic chromatin configurations, rather than through transcription factor activation alone.

**Figure 2:**
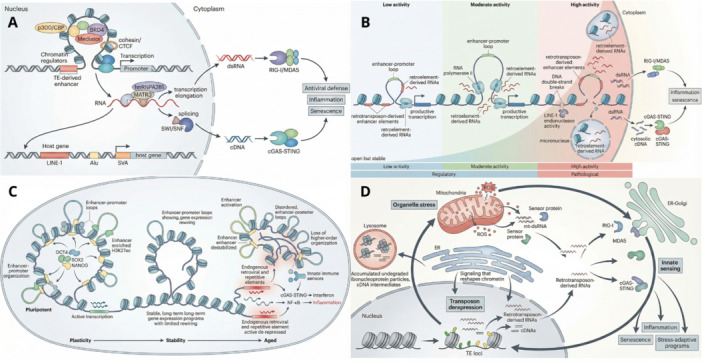
LINE‐1 biology, restriction, and genome‐modifying consequences relevant to TE‐mediated immune regulation. Figure 2 Legend. LINE‐1 (L1) elements provide a mechanistic example of how autonomous retrotransposons can generate regulatory, mutagenic, and immunostimulatory outputs. (A) A full‐length L1 element is transcribed into RNA, exported to the cytoplasm, translated into ORF1p and ORF2p, and assembled into an L1 ribonucleoprotein (RNP) complex. (B) During target‐primed reverse transcription, ORF2p endonuclease and reverse transcriptase activities enable genomic nicking, cDNA synthesis, and insertion of a new L1 copy, often accompanied by target‐site duplications. (C) L1 insertions can disrupt host gene structure by altering splicing, promoting exon skipping, or generating chimeric transcripts. (D) Host restriction pathways, including repressive chromatin marks such as H3K9me3 and small RNA‐associated silencing, limit L1 activity, whereas engineered systems such as CRISPR‐Cas9 or modified L1 RNP approaches illustrate how DNA breaks or retrotransposition‐related mechanisms can be experimentally redirected. This figure provides mechanistic context for the TE‐derived RNA/cDNA and genome‐modifying processes discussed in the TACTIR framework.

TE‐derived sequences provide a plausible evolutionary substrate for this layer of regulation because they can contribute binding sites for chromatin‐organizing proteins. Across human and mouse genomes, TE‐derived CTCF sites are enriched among cell‐ and species‐specific loop anchors, and TE‐associated looping variation is linked to differences in gene expression across cell types and species [98]. More broadly, comparative three‐dimensional genome analyses show that specific TE families and subfamilies have contributed to lineage‐specific loops and domain boundaries, with CRISPR‐Cas9 deletion of selected TE‐derived architectural elements disrupting local three‐dimensional chromatin structure [99]. Together, these findings are consistent with a restrained version of TACTIR in which selected TE‐derived loci may contribute to stimulus‐responsive enhancer activity, enhancer‐promoter contacts and local chromatin topology, while remaining mechanistically distinct from uncontrolled TE‐derived RNA or cDNA sensing. Figure 2.

## 3 Mechanistic Mapping of TACTIR

To clarify TACTIR, we distinguish two temporally and mechanistically distinct routes through which transposable elements can influence immune regulation. The first is an acute, cell‐intrinsic sensing route: when epigenetic repression, RNA processing or nucleic acid surveillance fails, TE‐derived nucleic acids can accumulate as immunostimulatory ligands [65]. In human THP‐1 monocytic cells, combined ADAR and hnRNPC deficiency exposes Alu‐containing endogenous dsRNA species that trigger MDA5‐dependent type I interferon responses [100]. In senescence‐associated contexts, LINE‐1 transcription can also generate LINE‐1 cDNA and promote cGAS‐STING‐dependent inflammatory programs [101]. These examples define direct TE‐derived nucleic acid sensing.

The second route operates through regulatory DNA and reflects longer‐term genomic co‐option rather than acute ligand sensing. In this route, TE insertions introduce or amplify transcription factor binding motifs and enhancer architectures that can later be engaged by cytokine‐ or inflammation‐responsive transcription factors. In human immune‐cell enhancers, primate‐specific ERVs and Alu elements introduced functional NF‐κB and IRF1 motifs, with evidence that some Alu‐derived NF‐κB sites underwent positive selection and increased predicted binding affinity in humans [80]. More broadly, multispecies analyses of TNF‐stimulated primary endothelial cells show that distinct TE subfamilies can contribute RELA/NF‐κB‐bound regulatory sites near inflammatory response genes, including a MER81‐derived enhancer whose deletion impaired TNF‐induced IFNGR2 expression [90]. Thus, TACTIR provides a framework for separating acute TE‐derived RNA/dsRNA/cDNA sensing from indirect TE‐mediated gene regulation: the former reflects derepressed nucleic acid accumulation sensed by innate immune pathways, whereas the latter reflects regulatory sequences that shape immune gene expression through motif integration, enhancer activity and chromatin architecture. Table 1.

**Table 1:** Mechanistic layers linking transposable elements to immune regulation.

Mechanistic layer	TE‐derived input	Principal pathway	Functional consequence	Representative evidence
Innate immune sensor activation	TE‐derived dsRNA; LINE‐1 RNA/cDNA; selected retroelement‐derived RNA: DNA hybrids or cDNA intermediates	RIG‐I/MDA5‐MAVS; cGAS‐STING	TE‐derived nucleic acids are normally constrained by epigenetic silencing, RNA processing, and nucleic acid surveillance systems. Loss of these controls can permit innate immune sensing, leading to type I interferon and inflammatory activation.	HUSH/MPP8‐dependent LINE‐1 control limits dsRNA‐driven RIG‐I/MDA5 activation; LINE‐1‐derived or retroelement‐derived cDNA can engage DNA‐sensing pathways [102, 103].
Cis‐regulatory immune control	LTRs and ERV‐, LINE‐, Alu/SINE‐derived regulatory DNA	Transcription factor motif integration; enhancer/promoter activity; chromatin regulation	Fixed TE insertions can modulate immune gene expression without requiring TE RNA or cDNA intermediates to act as immune ligands.	Selected TE sequences can act as cis‐regulatory substrates near immune genes and contribute regulatory elements or immune‐related transcription factor motifs [103].
Organelle/stress‐associated feedback	Stress‐associated TE RNA/cDNA output; LINE‐1 RNP or protein intermediates	Inflammatory signaling; STING‐dependent trafficking; endolysosomal degradation	Stress signaling may increase TE‐derived immunostimulatory nucleic acids in selected contexts, whereas organelle‐linked quality‐control pathways can restrict TE protein or RNP intermediates.	LPS has been associated with modulation of HERV/MaLR expression in human PBMCs; STING can restrict LINE‐1 by routing ORF1p toward lysosomal degradation [75, 104].

### 3.1 Clinical Significance

The clinical relevance of transposable elements should be framed across a spectrum of molecular involvement rather than as a single transposon‐driven disease mechanism. At one end of this spectrum, new TE insertions can directly disrupt coding exons, introns or regulatory regions, producing loss‐of‐function alleles through frameshifts, premature termination, exon skipping, intron retention or aberrant splice‐site usage. These events are biologically important because they provide clear examples of ongoing retrotransposition as a mutagenic process, but they remain quantitatively rare within most disease mutational spectra. Thus, hemophilia A should be presented as a disorder in which Alu or LINE‐1 insertions into F8 have been reported as pathogenic events, not as a disorder primarily caused by TE insertions; most cases are explained by other F8 alterations, including recurrent inversions, sequence variants and copy‐number changes. Similarly, in neurofibromatosis type 1 (NF1), Alu and LINE‐1 integrations can disrupt NF1 splicing, including deep intronic LINE‐1 insertions that cause exon skipping, but TE integrations account for only approximately 0.4% of NF1 mutations. This quantitative context preserves the mechanistic importance of TE‐mediated mutagenesis while avoiding overstatement of its relative contribution to disease etiology.

Pathogenic TE insertions often act by converting noncoding sequence into aberrant transcript structure. In NF1, partial LINE‐1 integration within an intron can introduce poly(A)‐rich sequence, target‐site duplication and local transcript disruption, ultimately altering exon recognition and producing exon skipping [105]. A similar principle applies to cancer predisposition genes: an AluSx‐like insertion in MLH1 intron 7 was shown to disrupt normal MLH1 splicing by introducing Alu‐derived sequence into the transcript and promoting aberrant termination at the Alu poly(A) region, providing a concrete example of how TE insertions can interfere with mismatch‐repair gene function in Lynch syndrome [106]. These examples support TE insertional mutagenesis as a clinically relevant but low‐frequency mechanism that should be distinguished from broader TE dysregulation.

In cancer, TE involvement is often better understood as a consequence and modifier of epigenetic instability rather than as purely insertional mutagenesis. Cancer‐associated DNA hypomethylation and altered chromatin repression can reactivate ERVs, LINE‐1 and other TE families, leading to TE‐derived promoters, enhancer‐like activity, aberrant transcripts, ORF1p/ORF2p expression and, in some tumors, new somatic retrotransposition.^107108^ However, even in cancer genomes where LINE‐1 mobilization is detectable, most insertions are likely passengers; only a small subset are considered tumor‐initiating events [107]. This distinction is important because TE expression can influence tumor biology without requiring every TE event to be a driver mutation. In colorectal cancer, TE overexpression has been associated with poor outcome, interferon pathway activation, immune infiltration and immune evasion, with PD‐L1 expression mainly localized to tumor‐associated immune cells rather than tumor cells themselves [108]. Thus, TE dysregulation may influence tumor‐immune architecture through transcriptional and immunological mechanisms even when insertional mutagenesis is not the dominant cause.

TE activity is also relevant to normal and pathological immune cell states. In the thymus, TE expression is not simply pathological noise: human and mouse thymic analyses show broad TE expression in medullary thymic epithelial cells and plasmacytoid dendritic cells, TE interactions with transcription factors involved in thymic development and function such as PAX1 and REL, and regulation of nonredundant TE sets by AIRE, FEZF2 and CHD4 [109]. These findings support a role for controlled TE expression in thymic antigen presentation, T‐cell selection and immune self‐tolerance [110]. In cancer‐associated T‐cell dysfunction, TE expression is likewise cell‐state‐dependent. Selective TE regulation has been reported in mouse and human CD8+ tumor‐infiltrating lymphocytes during exhaustion and anti‐PD‐1 treatment, including regulated VL30 expression in mouse exhausted T‐cell subsets and increased TE expression after immune checkpoint blockade [110, 111]. Together, these studies argue that TE regulation should be interpreted in relation to cellular state, lineage and immune context rather than treated as uniformly deleterious.

This disease model also integrates TE control, organelle stress and immune signaling. Epigenetic control helps determine whether TE loci remain constrained, become transcriptionally permissive or progress toward productive RNA/cDNA output. Organelle and stress pathways can alter this balance: mitochondrial dysfunction can increase LINE‐1 ORF1p expression and reduce DNA cytosine methylation [111] in neuronal disease models [73], Immune circuitry then interprets the resulting TE‐derived products in a state‐dependent manner. When repression and quality‐control systems remain intact, TE‐derived sequences may contribute to regulated chromatin or immune‐cell programs; when these systems fail, TE‐derived RNA, cDNA or RNP intermediates can feed viral mimicry‐like responses through pathways such as RIG‐I/MDA5‐MAVS or cGAS‐STING [107, 112]. This integrated view connects TE regulation, organelle stress and immune signaling without implying that all organelle stress responses are TE‐driven or that all TE activation is pathological.

LINE‐1 ribonucleoprotein particles provide a useful mechanistic and experimental bridge across these disease states. Because LINE‐1 ribonucleoprotein (RNP) assembly precedes productive retrotransposition, ORF1p/ORF2p measurements can help distinguish transcriptional derepression from mobilization competence [113]. RNP assembly precedes productive retrotransposition, LINE‐1 RNPs can be used experimentally to distinguish simple transcriptional derepression from mobilization competence or immune‐stimulatory nucleic acid production. Measurements of ORF1p/ORF2p abundance, localization, RNA association, enzymatic activity or lysosomal routing therefore provide practical entry points for mapping where TE control fails in a given disease context [75, 113].

Together, these disease examples support a calibrated interpretation of TE biology. Rare insertional events can cause monogenic disease or cancer predisposition; broader TE derepression can reshape cancer transcriptional and immune states; regulated TE expression can participate in thymic and T‐cell state biology; and organelle stress can either amplify or restrict TE‐derived RNA, cDNA and RNP intermediates. This view links TE regulation to disease without implying that TE insertions dominate every condition discussed.

## Data Availability

Data sharing not applicable to this article as no datasets were generated or analysed during the current study.
